# Risk factors for iliopsoas impingement after total hip arthroplasty using a collared femoral prosthesis

**DOI:** 10.1186/s13018-020-01787-3

**Published:** 2020-07-16

**Authors:** Jiandi Qiu, Xiurong Ke, Shanxi Chen, Liben Zhao, Fanghui Wu, Guojing Yang, Lei Zhang

**Affiliations:** 1 Department of Adult Reconstruction, The Third Affiliated Hospital of Wenzhou Medical University, Wenzhou, 325200 Zhejiang Province China; 2 Department of Radiology, The Third Affiliated Hospital of Wenzhou Medical University, Wenzhou, 325200 Zhejiang Province China; 3 Department of Sports Medicine, The Third Affiliated Hospital of Wenzhou Medical University, No.108, Wansong Road, Ruian, Wenzhou, 325200 Zhejiang China

**Keywords:** Iliopsoas impingement, Hip, Arthroplasty, Collared femoral prosthesis, Complication, Risk factor

## Abstract

**Background:**

The relationship between collar design of a femoral component and iliopsoas impingement (IPI) after total hip arthroplasty (THA) is still underrecognized. The purpose of our study was to determine the possible risk factors for IPI related to the femoral component, when using a collared femoral prosthesis.

**Methods:**

A total of 196 consecutive THA patients (206 hips) using a collared femoral prosthesis were reviewed retrospectively after exclusion of the factors related to acetabular component and femoral head. The patients were divided into +IPI and −IPI group according to the presence of IPI. Radiological evaluations were performed including femoral morphology, stem positioning, and collar protrusion length (CPL). Multivariate regression analysis was performed to assess the risk factors for IPI.

**Results:**

At a minimum follow-up of 1 year, IPI was observed in 15 hips (7.3%). Dorr type C proximal femur was found in nine hips (60%) in the +IPI group and in 28 hips in the −IPI group (14.7%, *p* < 0.001). The mean stem anteversion in the +IPI group was significantly greater than that in the −IPI group (19.1° vs. 15.2°, *p* < 0.001), as well as the mean CPL (2.6 mm vs. − 0.5 mm, *p* < 0.001). The increased stem anteversion (*OR* = 1.745, *p* = 0.001) and CPL (*OR* = 13.889, *p* = 0.001) were potential risk factors for IPI.

**Conclusions:**

The incidence of IPI after THA is higher than expected when using a collared femoral prosthesis. Among the factors related to collared femoral prosthesis, excessively increased stem anteversion and prominent collar protrusion are independent predictors for IPI. In addition, high risk of IPI should be carefully considered in Dorr type C bone, despite that femoral morphology is not a predictive factor.

**Level of evidence:**

Level IV, clinical cohort study

## Introduction

Iliopsoas impingement (IPI) is one of the underrecognized causes of hip pain after primary total hip arthroplasty (THA), which is characterized by persistent groin pain and physical disability [1, 2]. IPI after primary THA has considerable medical consequences and an incidence as high as 3.9–4.6% [3, 4]. Although the diagnosis of IPI remains a challenge, it is still meaningful to recognize the potential etiologies and thus to take proper precautions against it.

Among various factors contributing to IPI, the factors related to the acetabular component have been examined in previous studies, including cement extrusion [5], the penetrated screws for acetabular fixation [6] and a mal-positioned acetabular cup [7, 8], or the reinforcement ring [9]. Apart from the acetabular component, the liner and the femoral head have also been reported to lead to IPI [10, 11]. Very few studies have focused on the impingement secondary to the femoral prosthesis.

Even if the benefits of collars are unclear, they remain widely used, in several femoral stem designs, to be expected to improve immediate stability in THA [12]. Several national joint registries reported a significant trend in favor of collar use, showing collared stems had a statistically significant difference in risk of subsidence and peri-prosthetic fracture in comparison to collarless stems [13, 14]. As an underestimated factor, a collared femoral prosthesis has been regarded as an unusual cause of IPI in only two case reports [15, 16]. In these studies, the authors reported a limited number of patients with persistent groin pain, which was all caused by the impingement between the metal collar and the iliopsoas tendon as it overhung the calcar. To date, no previous studies have attempted to investigate relevant factors for IPI which are related to the femoral component, particularly when using a collared femoral prosthesis.

The primary purpose of our retrospective study was to determine the risk factors related to the femoral component that could potentially lead to IPI after primary THA using a collared femoral prosthesis, through radiological assessments. Furthermore, the secondary purpose was to investigate the possible mechanism of IPI and therefore to discuss effective preventive strategies.

## Methods

### Patient selection

Between September 2015 and December 2017, 267 consecutive hips in 257 patients, who underwent a THA using a collared femoral prosthesis and completed a minimum follow-up of 1 year, were retrospectively screened. The exclusion criteria included (1) malposition of the acetabular component after radiographic evaluations according to Lewinnek’s safe zone [17]; (2) severe hip dysplasia; (3) mental disorder including prolonged delirium and dementia, who were unable to precisely describe the symptoms; (4) low quality radiographs; (5) patients developing major complications within the follow-up period such as dislocation, infection, and loosening; and (6) incomplete data. The protocol was approved by the institutional review board at the hospital (MS20190728).

**Fig. 1:**
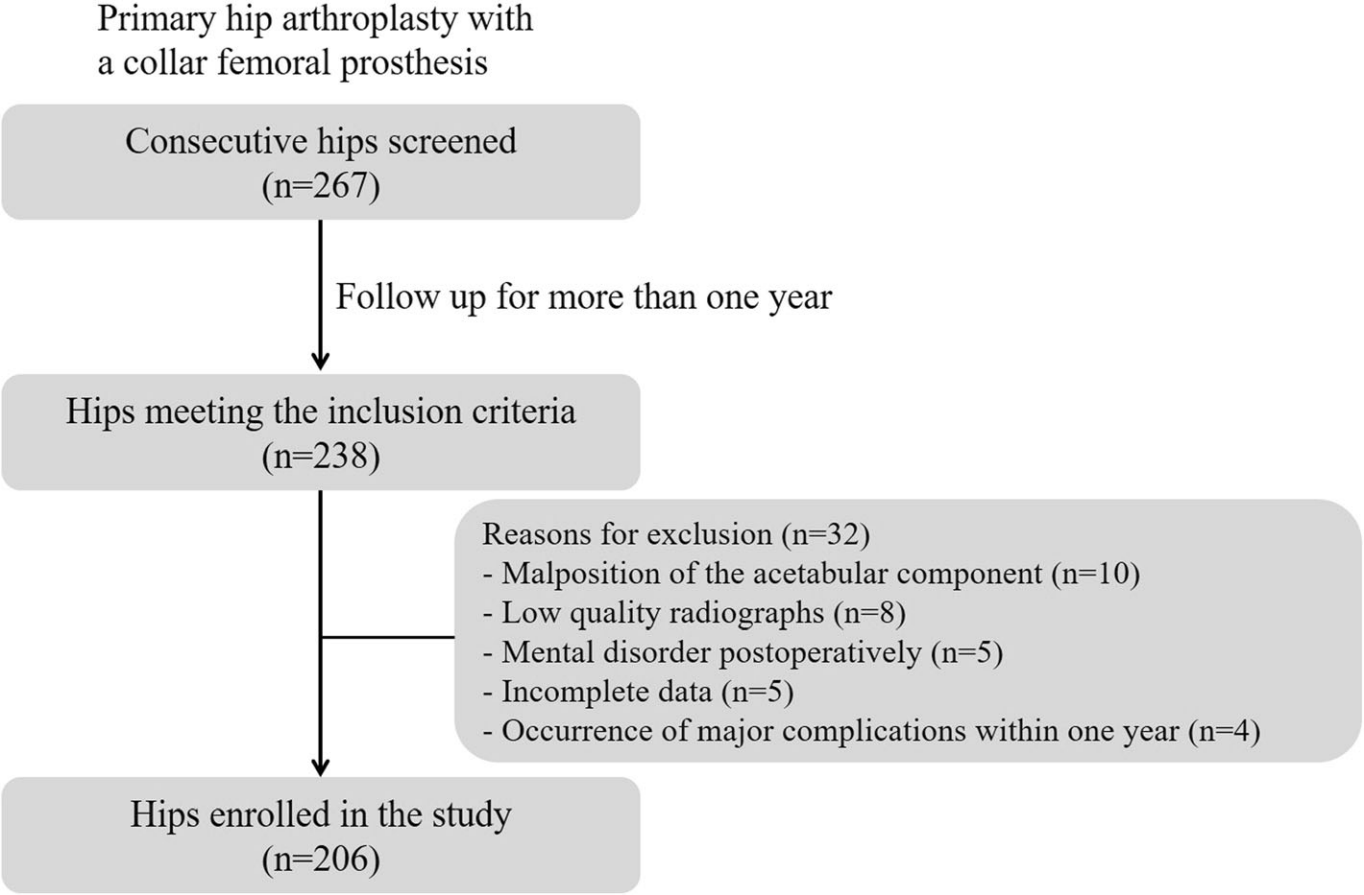
Flow chart of patient enrollment

Among 267 hips, 238 hips who met the inclusion criteria were eligible to be included in the study (Fig. 1). Subsequently, the exclusion criteria ruled out 32 hips, and the remaining 206 hips in 196 patients were enrolled in the final cohort.

### Diagnosis of IPI

The patients were classified into the +IPI and −IPI group according to the presence of IPI symptoms. Patient meeting the following criteria were diagnosed as IPI: (1) persistent anterior groin pain after 3 months postoperatively; (2) anterior groin pain triggered by active hip flexion and active flexion against resistance with pain continuing from 30 to 70° flexion; (3) pain increased in active internal rotation and reduced in external rotation; and (4) pain improved after injection with lidocaine and steroid into the iliopsoas tendon sheath under the guidance of ultrasound [1, 18]. The diagnosis of IPI was documented by a senior surgeon during the follow-up.

### Surgical technique

Uncemented THA was performed in all patients via a posterolateral approach, using a Pinnacle® acetabular cup system (Depuy, Warsaw, IN, USA) on the acetabular side and a Corail® femoral stem system with collar design (Depuy, Warsaw, IN, USA) on the femoral side. Accordingly, a 32 mm or 36 mm ceramic femoral head was used. Surgical efforts were made to achieve equal leg length, proper stem alignment, optimal hip offset, and axial and rotational stability of the prosthesis. We did not perform iliopsoas tendon release at level of the lesser trochanter. No major complications were identified intraoperatively. A standardized rehabilitation protocol was applied in collaboration with a physiotherapist. Physiotherapy began as soon as the anesthetic had resolved and was monitored during the whole process. Physiotherapy sessions initially emphasized bed transfers, movement from sitting to standing, and then processed to weight-bearing activities as tolerated within 24 h postoperatively, with the assistance of a walking aid. In addition, the patients were instructed in a home-based rehabilitation program emphasizing recovery of muscle strength and range of motion of the joints.

### Radiographic evaluations

All patients were reviewed postoperatively at 6 weeks, 3 and 6 months, then followed by yearly reviews thereafter. The baseline characteristics were documented including age, gender, body mass index, and diagnoses.

All radiographs were taken by a senior radiologist in a standardized manner. Radiologic measurements were performed using three standard views of X-ray: pelvic anteroposterior (AP) view, AP view, and true lateral view of the operated femur. To minimize potential magnification errors, a metal coin of a known diameter was used as the reference on the AP view of the operated femur. We evaluated on these X-rays to exclude major complications such as prosthesis loosening, malposition of the acetabular component, and periprosthetic fracture.

The morphology of the proximal femur was categorized on the preoperative AP view of the femur as described by Dorr et al. [19]. We used the canal-to-calcar ratio (CCR) for classification. Femurs with CCR of 0–0.500 were categorized as Dorr type A, 0.501–0.750 as type B, and 0.751–1.000 as type C. The intraclass correlation coefficient (ICC) for intraobserver agreement was 91.4% (95% confidence interval, 88.5–94.3%), and ICC for interobserver agreement was 88.7% (95% confidence interval, 85.2–92.2%).

**Fig. 2:**
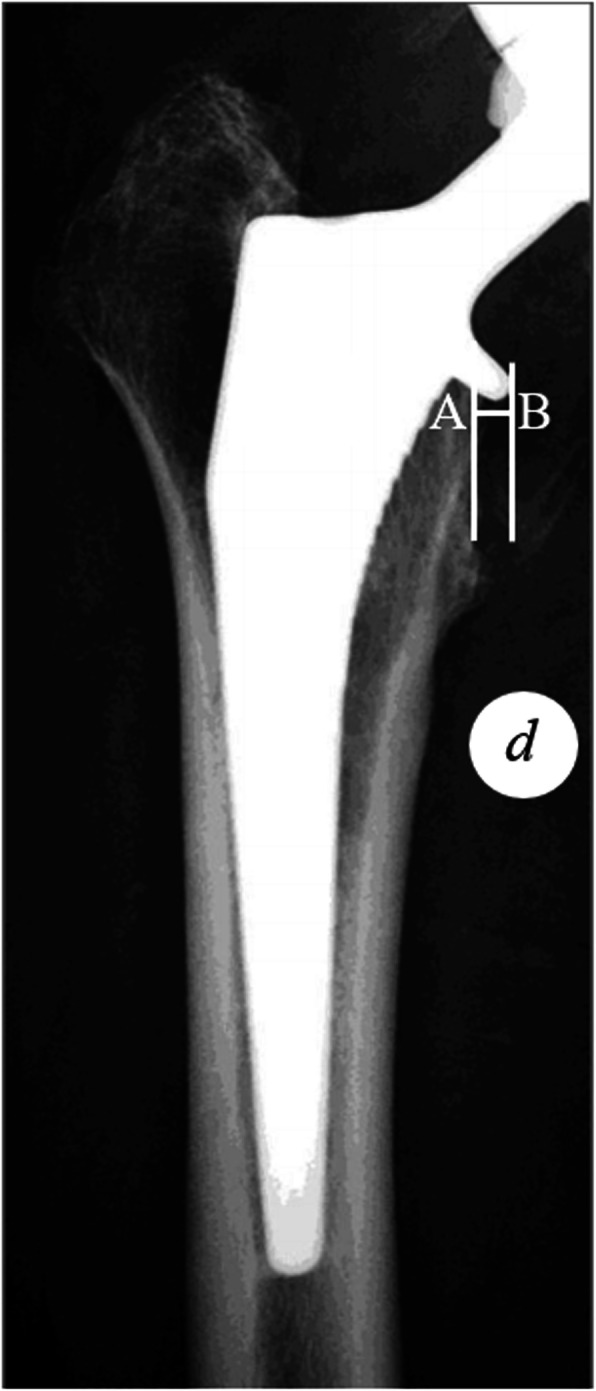
Measurement of collar protrusion length (CPL). Point A represents the medial aspect of the calcar and point B represents the medial aspect of the metal collar. *d* represents the diameter of coin measured on the X-ray. CPL was measured as the distance between the vertical line across points A and B, after correction for magnification. A positive CPL represents overhang of the collar over the calcar

Stem alignment was evaluated on postoperative AP radiograph of the femur, according to the intersection angle between vertical axis of the stem and diaphyseal femoral axis. A positive value represents valgus alignment of the femoral stem, and vice versa. Furthermore, we introduced a variable termed collar protrusion length (CPL) to evaluate the severity of collar protrusion. On the postoperative AP radiograph, CPL was measured as the distance between the medial aspect of the metal collar and the medial aspect of the calcar (Fig. 2). A true CPL can be obtained after correcting the magnification error. In terms of CPL, a positive value represents an overhang of the collar, and a negative value represents that the collar stays within the calcar region.

**Fig. 3:**
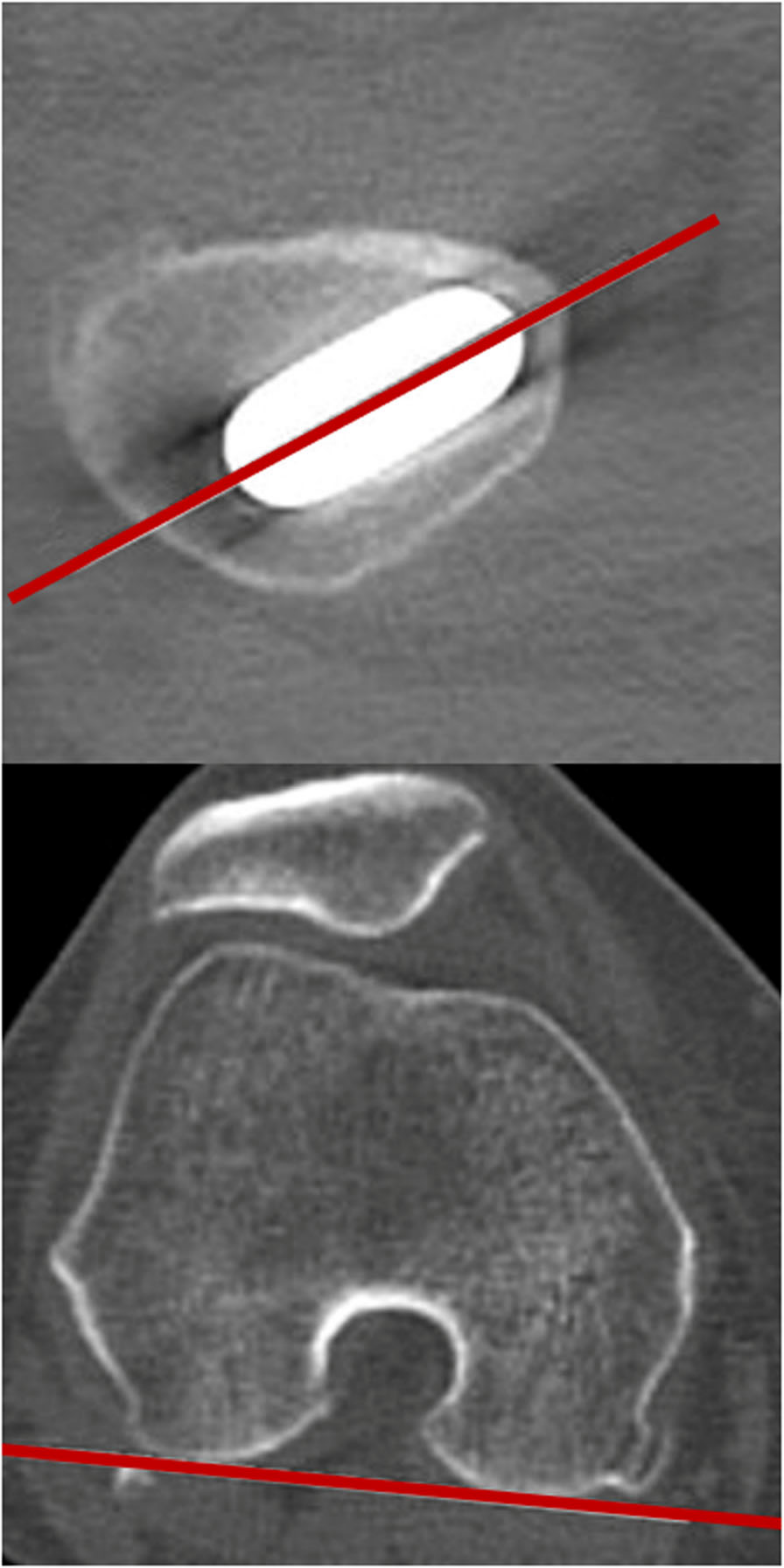
Measurement of stem anteversion on CT scan. The anteversion angle was measured as the angle between the stem-neck axis and an axis parallel to the posterior aspect of the femoral condyles. After measurement, the anteversion angle of the stem was 35°

We evaluated anteversion of the femoral stem on axial CT scans taken with the patient in a supine position, from the pelvis to distal femur, using a metal artifact removal method. The anteversion angle was measured as the angle between the stem-neck axis and an axis parallel to the posterior aspect of the femoral condyles, measured in the transverse plane (Fig. 3) [20].

The radiological measurements of stem alignment, CPL, and anteversion were performed by one experienced orthopedic surgeon in a blinded manner. These measurements were repeated twice on separate occasions, and the values were averaged.

### Statistical analysis

Statistical analyses were performed using SPSS version 19.0 (SPSS Inc., Chicago, Illinois). Univariate analyses were performed to identify the potential risk factors for IPI between the two groups. In the univariate analyses, quantitative data were compared by Student’s *t* test, and categorical variables were compared by Pearson chi-square test, continuity correction, or Fisher’s exact test. Subsequently, the statistically significant variables were selected for inclusion in a multivariate logistic regression model, using a stepwise method. Odds ratio (*OR*) was used to describe proportionate risks of IPI. A *p* value < 0.05 was considered significant. Additionally, overall calibration of the regression model was tested using a Hosmer-Lemeshow goodness of fit test.

## Results

Out of 206 hips, 15 hips (15 patients) had IPI, and the incidence was 7.3%. In terms of baseline data, no statistical differences were observed between the +IPI and −IPI groups (Table 1).

**Table 1:** Baseline characteristics in the iliopsoas impingement (+IPI) and non-impingement (−IPI) group

	+IPI group (*n* = 15)	−IPI group (*n* = 191)	*P* value
Number of patients	15	181	
Gender, no. (%)			0.420
Female	9	89	
Male	6	92	
Age (years)			0.453
Mean (SD)	69.7 (10.1)	67.9 (9.0)	
Median (range)	68 (55–88)	68 (45–91)	
BMI (Kg/m^2^)			0.359
Mean (SD)	24.3 (4.2)	25.3 (19.0)	
Median (range)	24.5 (16.5–33.1)	25.1 (17.7–32.9)	
Diseases, no. (%)			0.094^a^
FNF	5	40	
ONFH	3	93	
DDH	6	53	
OA	1	5	

*BMI* body mass index, *FNF* femoral neck fracture, *ONFH* osteonecrosis of the femoral head, *DDH* developmental dysplasia of the hip, *OA* osteoarthritis, *SD* standard deviation

^a^Fisher exact test

The radiological variables of these 15 patients are listed in Additional file 1. In the +IPI group, 9 hips (60%) were identified with Dorr type C femur, whereas only 28 hips (14.7%) in the −IPI group (Table 2). A significant difference was observed between these two groups regarding proximal femoral morphology (χ^2^ = 14.36, *p* < 0.001).

**Table 2:** Radiological variables in the +IPI and −IPI group

	+IPI group (*n* = 15)	−IPI group (*n* = 191)	*P* value
Number of patients	15	181	
Dorr classification, no. (%)^*^			< 0.001^a^
Type A	2 (13.3)	54 (28.3)	
Type B	4 (26.7)	109 (57.1)	
Type C	9 (60)	28 (14.6)	
Stem alignment (°)			0.065
Mean (SD)	1.2 (1.9)	0.4 (1.6)	
Median (range)	1 (− 1–3)	0 (− 2–3)	
Stem anteversion (°)*			< 0.001
Mean (SD)	19.1 (8.9)	15.2 (2.9)	
Median (range)	16 (10–39)	15 (9–25)	
CPL (mm)^*^			< 0.001
Mean (SD)	2.6 (2.3)	− 0.5 (1.1)	
Median (range)	3 (− 2–5)	0 (-2-1)	

*CPL* collar protrusion length, *SD* standard deviation

^*^Difference of data statistically significant (*P* < 0.05)

^a^Fisher exact test

We did not observe severe stem malalignment in all the patients (Table 2). A slightly varus and valgus alignment were acceptable (− 2 to 3°) with no statistical difference between the two groups (*t* = 1.857, *p* = 0.065).

The mean stem anteversion in the +IPI group was 19.1°, significantly greater than that in the −IPI group (15.2°, *t* = 4.003, *p* < 0.001) (Table 2). In the +IPI group, the anteversion angle > 30° was found in 3 hips (32°, 35°, 39°, respectively), which was absent in the −IPI group.

Similarly, a greater CPL was observed in the +IPI group (2.6 ± 2.3 mm) when compared to the −IPI group (− 0.5 ± 1.1 mm; *t* = 9.538, *p* < 0.001) (Table 2). In the +IPI group, collar protrusion was found in 13 hips (86.7%), whereas 40 hips (20.9%) in the −IPI group, showing a statistical difference between the two groups (*p* < 0.001).

The multivariate logistical regression determined increased stem anteversion (*OR* = 1.745, *p* = 0.001) and CPL (*OR* = 13.889, *p* = 0.001) as the risk factors for IPI (Table 3). Moreover, the Hosmer-Lemeshow goodness-of-fit test indicated that the regression model offered a good fit for the data (*p* = 0.959). The number needed to treat (NNT) with Dorr type C and positive CPL to cause one IPI was 4.8 patients and 4.7 patients, respectively.

**Table 3:** Results of logistic regression model to predict iliopsoas impingement

Multivariate predictor	Regression coefficient	OR	95% CI	***P*** value
Primary disease	− 0.349	0.705	0.161–3.095	0.643
Femoral morphology	0.766	2.151	0.447–10.309	0.339
Stem alignment	0.129	0.879	0.362–2.136	0.776
Stem anteversion*	0.556	1.745	1.250–2.433	0.001
CPL*	2.638	13.889	3.145–62.500	0.001

*OR* odds ratio, *CI* confidence intervals, *CPL* collar protrusion length

*Difference of data statistically significant (*P* < 0.05)

## Discussion

Most recently, there has been an increased trend towards the use of collared femoral stems in preference to collarless stems, due to reported superiority in lowering risks of subsidence and intraoperative fractures [21, 22]. The present study retrospectively revealed a high incidence of IPI (7.3%) in THA patients using a collared femoral prosthesis. After excluding factors related to the acetabular component and the femoral head, our study identified increased stem anteversion and CPL as the risk factors for IPI. Although femoral morphology was not a predictive factor, patients with Dorr type C bone appeared with higher risk of IPI after THA, when compared to those with Dorr types A and B.

The strength of the present study is that our design provided the most efficient scheme for rare factors with sufficient sample size because it only focused on the effect of the femoral component on IPI, after ruling out the effect of the acetabular component, the liner, and the femoral head. Only two case reports documented several cases of IPI when using a collared femoral prosthesis [15, 16]. They failed to provide convincible evidences regarding the associated effect of the collar on impingement due to their limited cases. To our knowledge, no previous studies have made such in-depth analyses of relevant factors for IPI. As a step towards exploring the definitive risk factors for IPI, we finally employed this cohort study to highlight the significance for clinical practice.

Successful THA depends to a great extent on ideal orientation of the acetabular and femoral components. Concerning the independent effect of the femoral component, high variability of femoral anteversion can be found after primary THA using a standard uncemented collarless stem, but it did not impact on the final clinical outcomes [23]. However, the conflicting results can be reached when using a collared femoral stem. The present study observed that increased anteversion of a collared femoral prosthesis resulted in high incidence of IPI. Positioning a collared femoral stem at an excessively increased anteversion may cause overhang of the collar beyond the edge of the calcar, and ultimately result in impingement on the distal segment of the iliopsoas tendon at level of the lesser trochanter. It could explain the reason why greater CPL was another risk factor for IPI because the increased CPL was frequently associated with increased stem anteversion. In agreement with our results, several previous studies reported a limited number of IPI cases associated with collar protrusion. Brew et al. reported an unusual case of iliopsoas tendonitis caused by protrusion of the collar on a femoral prosthesis [15]. Conversion of the stem to a collarless prosthesis finally led to immediate and complete relief of groin pain. In another case report by Lindner et al., IPI was found in a patient undergoing THA with a collared femoral stem due to a 13-mm overhang of the collar over the femoral calcar and was successfully treated by endoscopic iliopsoas tenotomy [16]. Modified strategies regarding hip implant selection and surgical techniques should be reconsidered in selected cases when using a collared femoral prosthesis, aiming to avoid this underestimated complication. As described below, use of collarless stem, appropriate level of the femoral neck cut, or avoiding excessive anteversion of a collared stem might be an effective and reliable treatment option for patients at high risk for IPI.

Uncemented femoral component, regardless of collared or uncollared, can provide primary stability and achieve favorable clinical outcomes in Dorr type C bone [24–26]. However, the relationship between IPI and proximal femoral morphology has not been well established. Our study did not observe femoral morphology as a predictor for IPI. Even though, we observed statistically higher incidence of IPI in Dorr type C bone (9/37, 24.3%) when compared to Dorr types A (3.6 %) and B (3.5 %). In contrast to Dorr types A and B femurs, Dorr type C femur commonly indicates a requirement of bigger size stem. Bonin et al. digitally analyzed CT scans of 204 healthy hips and found that according to native proximal femoral anatomy, a single collar size was not sufficient to ensure outstanding performance of collared stems [27]. Therefore, as an increase in stem size is inevitably associated with the increases in collar length, it can explain in our study why a majority of IPI were observed in Dorr type C femur.

**Fig. 4:**
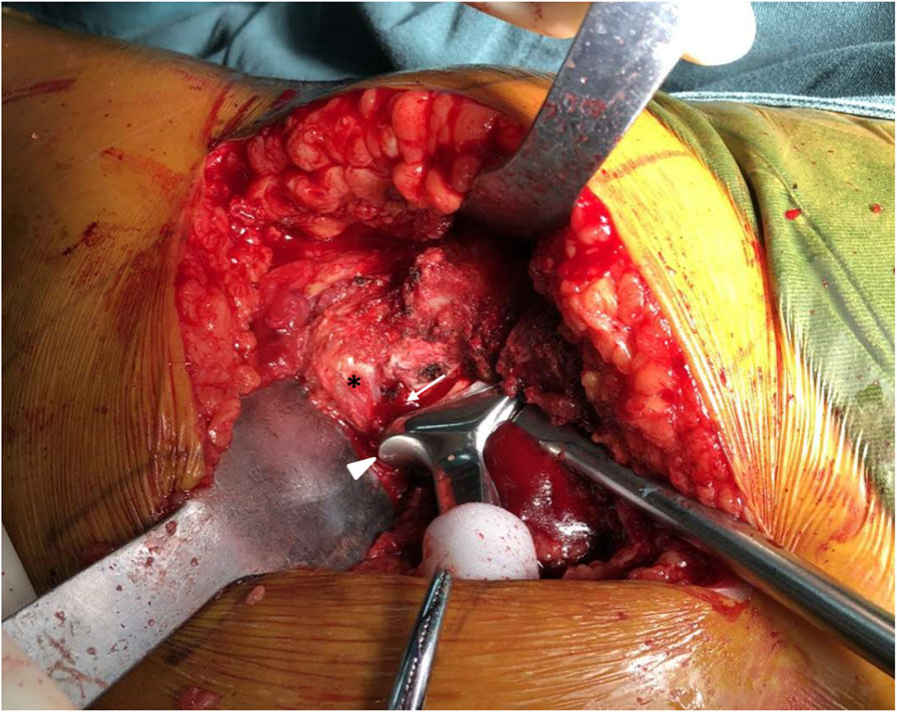
Intraoperative photo demonstrating potential impingement between the collar (white arrowhead) and iliopsoas tendon (black star) during stem implantation. Note that the metal collar was overhanging the calcar (white arrow) even if the stem had not been fully seated yet into the canal

The present study indicated that in addition to the acetabular component, we should place particular attention to the femoral component-related factors contributing to IPI when using a collared femoral prosthesis. To avoid IPI, the surgeons should take these risk factors into consideration. One important thing needed to be emphasized is the accurate orientation of the femoral component, particularly in Dorr type C femur. During THA, even if the acetabular component is placed in less anteversion, the femoral stem should not be regulated in excessively increased anteversion when using a collared femoral prosthesis, to obtain a proper combined anteversion [28], because the increased anteversion of the femoral stem might potentially lead to overhang of the collar and thereby cause impingement of the iliopsoas tendon on the collar during hip flexion. Furthermore, the surgeons should be aware of the impingement resulted from a low femoral neck cut, especially in osteoporotic patients. If the femoral neck is cut too low and close to the lesser trochanter, there is higher risk of impingement against the iliopsoas tendon near its insertion (Fig. 4). To obtain required leg length and offset, the solutions for these problems are high level of the femoral neck cut, using a shorter modular head or using a collarless stem [6].

There are certain limitations to our study. First, the retrospective scheme is the main limitation of our study. Randomized controlled trials are needed in the future to generate higher level of evidence. Second, although patients with acetabular component malposition were ruled out, the impingement in correct acetabular component position cannot be fully excluded. Moreover, not all of the impingement-related factors have been investigated yet including femoral head size [29]. We acknowledge the possibility of selection bias regarding the patient enrollment. Further studies are needed to investigate the combined factors related to both the acetabular and femoral components. Third, our study did not investigate clinical outcomes of the two groups, such as patient satisfaction and functional scores. Finally, IPI occasionally causes minor symptoms and resume most normal daily activities. There could also be the possibility that unrecognized groin pain was ignored by the family members and inexperienced surgeons at early stage of the study. Therefore, we still acknowledged a small number of missing patients as one of the weakness of our study.

## Conclusion

The incidence of IPI after THA is higher than expected, especially when using a collared femoral prosthesis. Among the factors related to collared femoral prosthesis, excessively increased stem anteversion and prominent collar protrusion are independent predictors for IPI. In addition, high risk of IPI should be carefully considered in Dorr type C bone, despite that femoral morphology is not a predictive factor. Further detailed studies to confirm these will be required in the future.

## Supplementary information

**Additional file 1.** The Baseline characteristics and radiographic variables of 15 patients in the iliopsoas impingement (+IPI) group.

## Data Availability

The data and materials are available from the department of Adult Reconstruction, The Third Affiliated Hospital of Wenzhou Medical University. The datasets used and analyzed during the current study are available from the corresponding author on reasonable request.

## Abbreviations

IPI: Iliopsoas impingement
THA: Total hip arthroplasty
CPL: Collar protrusion length
AP: Anteroposterior
CCR: Canal-to-calcar ratio
ICC: Intraclass correlation coefficient
OR: Odds ratio
BMI: Body mass index
FNF: Femoral neck fracture
ONFH: Osteonecrosis of the femoral head
DDH: Developmental dysplasia of the hip
OA: Osteoarthritis
SD: Standard deviation
CI: Confidence intervals
