# Supplementary material for: Risk factors for iliopsoas impingement after total hip arthroplasty using a collared femoral prosthesis
Source: J Orthop Surg Res. 2020 Jul 16;15:267. doi: 10.1186/s13018-020-01787-3 (PMC7364523; doi:10.1186/s13018-020-01787-3)
Supplement: Supplementary file 1 — Additional file 1. The Baseline characteristics and radiographic variables of 15 patients in the iliopsoas impingement (+IPI) group. [file 13018_2020_1787_MOESM1_ESM.docx]

**Additional file**

The Baseline characteristics and radiographic variables of 15 patients in the iliopsoas impingement (+IPI) group.

| Patient no. | Gender | Age  (years) | BMI  (Kg/m^2^) | Disease | Femoral morphology ^a^ | Stem alignment  (degrees) | Stem anteversion  (degrees) | CPL  (mm) |
| --- | --- | --- | --- | --- | --- | --- | --- | --- |
| 1 | Female | 55 | 24.9 | DDH | Type B | 2 | 39 | -2 |
| 2 | Male | 58 | 26.4 | DDH | Type A | 0 | 35 | -2 |
| 3 | Female | 62 | 23.5 | ONFH | Type A | 3 | 15 | 3 |
| 4 | Male | 63 | 20.7 | ONFH | Type B | 3 | 14 | 5 |
| 5 | Female | 63 | 23.5 | OA | Type C | 1 | 14 | 3 |
| 6 | Male | 64 | 20.5 | ONFH | Type B | 0 | 32 | 3 |
| 7 | Female | 67 | 28.7 | FNF | Type B | 1 | 13 | 2 |
| 8 | Female | 68 | 24.3 | DDH | Type B | 0 | 19 | 5 |
| 9 | Male | 69 | 24.8 | DDH | Type A | 1 | 12 | 4 |
| 10 | Female | 69 | 16.5 | DDH | Type C | 0 | 17 | 0 |
| 11 | Male | 71 | 24.5 | DDH | Type C | 2 | 17 | 3 |
| 12 | Female | 80 | 26.2 | FNF | Type C | -1 | 10 | 5 |
| 13 | Female | 84 | 33.1 | FNF | Type A | 3 | 14 | 4 |
| 14 | Female | 85 | 18.0 | FNF | Type C | 0 | 19 | 4 |
| 15 | Male | 88 | 28.2 | FNF | Type C | 3 | 16 | 2 |

BMI, body mass index; FNF, femoral neck fracture; ONFH, osteonecrosis of the femoral head; DDH, developmental dysplasia of the hip; OA, osteoarthritis; CPL, collar protrusion length.

^a^ according to Dorr classification
